# Propionic Acid Supports the Development of Oocytes Derived from Bovine Early Antral Follicles

**DOI:** 10.3390/vetsci13060533

**Published:** 2026-05-29

**Authors:** Takuya Ui, Kota Ushiroshoji, Genya Ito, Tatsuo Noguchi, Tomomi Tadokoro, Koumei Shirasuna, Hisataka Iwata

**Affiliations:** 1 Department of Animal Science, Tokyo University of Agriculture, Funako 1737, Atsugi City 243-0034, Japan; takuyaui.0327@gmail.com (T.U.); 11526001@nodai.ac.jp (K.U.); 11426001@nodai.ac.jp (G.I.); t3noguch@nodai.ac.jp (T.N.); ks205312@nodai.ac.jp (K.S.); 2 HIsataka Iwata Genome Research Center, Tokyo University of Agriculture, Tokyo 156-8502, Japan; tt208848@nodai.ac.jp

**Keywords:** early antral follicles, propionic acid, oocyte growth, lipid, gene expression

## Abstract

The present study investigated the effects of propionic acid (PA) supplementation during the in vitro growth of oocytes derived from bovine early antral follicles (EAFs) on oocyte quality and granulosa cell (GC) characteristics. Oocyte–GC complexes (OGCs) were collected from bovine ovaries and cultured for 14 days in medium supplemented with or without 0.01 mM PA. Oocytes and GCs obtained from large antral follicles were used as in vivo controls. Oocytes cultured with PA exhibited higher nuclear maturation and fertilization rates than those cultured without PA. PA supplementation increased lipid content and ATP levels in both oocytes and GCs and enhanced glucose consumption in OGCs. However, compared with in vivo-grown oocytes, in vitro-grown oocytes showed lower ATP levels, lipid content, mitochondrial membrane potential, and fertilization ability. RNA-seq followed by K-medoids clustering analysis revealed that PA-induced genes in GCs showing expression patterns similar to those in vivo were mainly associated with oxidative phosphorylation, whereas genes showing expression patterns distinct from the in vivo profile were associated with the PI3K–Akt signaling pathway. In conclusion, PA improves the quality of in vitro-grown oocytes and is associated with enhanced energy status in both oocytes and GCs. However, some PA-induced gene expression patterns in GCs differed from those observed in vivo.

## Simple Summary

Volatile fatty acids are major energy sources in ruminants, and propionic acid (PA) is one of the most important volatile fatty acids. This study investigated the effects of PA on the in vitro growth of oocytes derived from bovine early antral follicles. In addition, the properties of oocytes and granulosa cells (GCs) cultured in vitro were compared with those developed in vivo. The results showed that PA supplementation improved oocyte nuclear maturation and fertilization rates. Although oocyte size and GC number did not differ between the PA-treated and control groups, PA increased lipid content and ATP levels in both oocytes and GCs and enhanced glucose utilization in oocyte–GC complexes. However, compared with in vivo-grown oocytes, in vitro-grown oocytes exhibited lower ATP levels, lipid content, mitochondrial membrane potential, and fertilization rates. RNA sequencing analysis of GCs revealed that PA altered the expression of genes associated with oxidative phosphorylation and the PI3K–Akt signaling pathway. Collectively, these findings suggest that PA affects the in vitro growth of bovine oocytes by modulating the physiological characteristics of GCs.

## 1. Introduction

Ruminants possess a unique metabolic system in which volatile fatty acids (VFAs) serve as major energy sources. The principal VFAs are acetic acid, propionic acid (PA), and butyric acid, which are produced through ruminal fermentation and are present at high concentrations (~122 mM) in the rumen fluid. Among these VFAs in rumen fluid, acetic acid is the most abundant (60–65%), followed by PA (20–25%) [1,2]. Acetic acid has been reported to be present at concentrations of 1–2 mM in blood and 0.19–0.38 mM in follicular fluid (FF) [3], suggesting that a portion of circulating VFAs is transferred into FF. Because FF provides the microenvironment for oocyte growth, attempts to improve in vitro culture conditions have focused on mimicking its composition. Previous studies demonstrated that supplementation of in vitro maturation (IVM) medium with acetic acid (1 mM) improved embryonic development while reducing mitochondrial membrane potential and lipid content in porcine oocytes [4]. These findings suggest that VFAs may influence oocyte growth under physiological conditions through metabolic regulation; however, the effects of VFAs on oocyte development remain poorly understood. PA is a three-carbon short-chain fatty acid and the second most abundant VFA in ruminants. Despite its physiological importance, few studies have investigated the effects of PA on oocytes and embryos, with most previous research focusing on neural cells and autism-related models. Previous studies demonstrated the relationship between PA and ruminal epithelial function and hepatic metabolism in cows [5,6,7]. In recent decades, in vitro embryo production has become widely used in both humans and cattle. However, the availability of oocytes is limited because only a small number of antral follicles can be obtained. Therefore, establishing an efficient culture system for oocytes derived from early-stage follicles, such as early antral follicles (EAFs), is essential. However, current in vitro culture systems remain suboptimal, and the quality of in vitro-grown oocytes is still inferior to that of in vivo-grown oocytes, suggesting that important regulatory factors are lacking under in vitro conditions.

In the present study, oocyte–granulosa cell complexes (OGCs) were collected from bovine EAFs and cultured in vitro with or without PA supplementation. The developmental competence and metabolic characteristics of the resulting oocytes were evaluated. In addition, RNA-seq analysis was performed using GCs obtained from OGCs cultured with or without PA, as well as GCs collected from antral follicles (AFs).

## 2. Materials and Methods

### 2.1. Experimental Design and Strategies

To investigate how PA affects oocyte quality and energy status through alterations in granulosa cell (GC) characteristics, we first examined the effects of PA on oocyte growth and developmental competence. Subsequently, the energy status of oocytes and GCs was evaluated, followed by comprehensive gene expression analysis of GCs.

First, oocyte growth and developmental competence were compared among oocytes grown in vitro (IVG group), oocytes grown in vitro with PA supplementation (IVG + PA group), and fully grown oocytes collected from large AFs (in vivo group). OGCs derived from EAFs were cultured with or without PA (0.01 mM) for 14 days. After culture, the rates of antrum formation and GC survival, total GC number, and oocyte diameter were evaluated. In addition, the oocytes were subjected to in vitro maturation (IVM) followed by fertilization to assess maturation and fertilization rates.

A total of 900 OGCs were cultured. Among them, oocytes derived from 420 OGCs were randomly selected for evaluation of growth parameters and other experiments. In vitro-grown oocytes derived from 300 OGCs and the corresponding in vivo oocytes were subjected to IVM, and randomly selected oocytes were used for assessment of nuclear maturation or subsequent experiments. Furthermore, oocytes derived from 180 OGCs and the corresponding in vivo oocytes were subjected to IVM followed by fertilization to evaluate fertilization rates. Spent culture media were collected at the end of the culture period and stored at −80 °C until analysis.

Second, energy status markers in oocytes and GCs were compared among the IVG, IVG + PA, and in vivo groups. In vitro-grown or in vitro-matured oocytes were analyzed for mitochondrial membrane potential, ATP content, mitochondrial DNA copy number (mt-cn), and lipid content. In addition, randomly selected spent culture media were used to assess glucose consumption. The number of oocytes and media samples used for each analysis is described in the corresponding figure legends.

Finally, gene expression profiles were compared among GCs derived from the IVG, IVG + PA, and in vivo groups using RNA sequencing (RNA-seq). For each group, 10 OGCs were cultured per replicate, and three biological replicates were performed (total = 30 OGCs/group). GCs collected from a total of 18 antrum-forming OGCs were subjected to RNA-seq analysis. GCs collected from AFs of the corresponding ovaries were also used as in vivo GCs.

### 2.2. Chemicals and Media

Unless otherwise stated, all chemicals were purchased from Nacalai Tesque (Kyoto, Japan). The medium used for in vitro growth (IVG) of oocytes was α-MEM (Sigma-Aldrich, St. Louis, MO, USA) modified as described previously [8,9]. Briefly, the medium contained 4.0 mM hypoxanthine (Sigma-Aldrich), 4% polyvinylpyrrolidone (K-90), 11 mM glucose, 0.9 mM sodium pyruvate, 0.05 μM dexamethasone, 50 µg/mL ascorbic acid, 55 µg/mL L-cysteine, 5% fetal calf serum, 0.02 mAU/mL follicle-stimulating hormone (Kyoritsu Seiyaku, Tokyo, Japan), 1 µg/mL 17β-estradiol (Sigma-Aldrich), and penicillin and streptomycin. In vitro maturation (IVM) of oocytes was conducted in mTCM-199 (Gibco BRL, Paisley, UK) medium containing 10% FCS, 10 IU/mL human chorionic gonadotropin (Fuji Pharma Co., Ltd., Tokyo, Japan), 10 ng/mL EGF, and 5 mM taurine (IVM medium). The medium used for in vitro fertilization (IVF) was synthetic oviduct fluid (SOF) modified from a previous report (IVF medium) [10].

GCs incubation, and collection and handling of oocytes were conducted in TCM-199 medium containing 5% FCS (IVC-medium).

### 2.3. Propionic Acid Concentration

In the preliminary experiment, plasma was collected from eight Japanese Black cows of the same age (four grain-fed cows, 750–800 kg, 2–3 years old; four grass-fed cows, 350–380 kg, 2–3 years old). These cows were housed at a university farm (grass-fed: free access to water and grazing; grain-fed: free access to water and grain feed in pens). Four plasma samples from the two categories were mixed equally to create two samples that were sent to Shimadzu Techno Research (Kyoto, Japan) to measure short-chain fatty acids. Acetic acid concentrations were 0.85 and 0.84 mM for grain-fed and grass-fed cows, respectively, and PA concentrations were 0.037 and 0.039 mM in grain and grass-fed cows, respectively. Based on the concentration and a previous report [3] showing that one third of the blood concentration was found in FF, 0.01 mM PA was used in the experiments. Furthermore, IVG medium supplemented with 0 (control) and 0.01 mM PA (0.01 mM) were incubated for 12 h and 14 days in 5% CO_2_ and 95% air at 38.5 °C, and the pH values of the media were 7.53 ± 0.01 (*n* = 3) and 7.51 ± 0.02 (*n* = 3) for 12 h and 7.34 ± 0.04 (*n* = 3) and 7.41 ± 0.07 (*n* = 3) at the end of incubation, respectively.

### 2.4. Collection of OGCs from EAFs and Oocytes and GCs from Large Antral Follicles (AFs)

**Figure 1:**
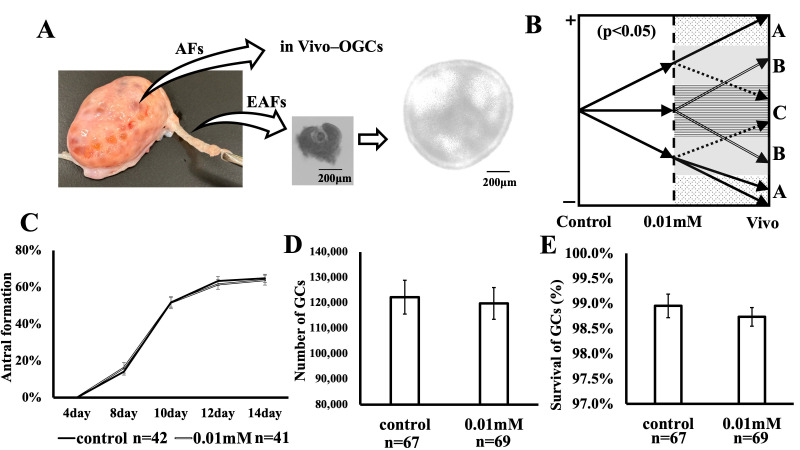
Methodological illustration of OGC collection, clustering analysis, and development of OGCs. (**A**) Collection of oocyte–granulosa cell complexes (OGCs) from early antral follicles (EAFs) and AFs. Representative images show an OGC immediately after collection from EAFs and an OGC forming an antrum during culture. Scale bar = 200 μm. (**B**) Schematic diagram of gene clustering analysis. Differentially expressed genes (DEGs) between the control and 0.01 mM PA groups (*p* < 0.05) were classified into eight clusters using K-medoids clustering. The clusters were further categorized into three groups according to similarity to in vivo granulosa cell (GC) gene expression profiles. (**C**) Rate of antrum formation in OGCs during culture (Student’s *t*-test). (**D**,**E**) Number (Kruskal–Wallis) and survival rate (Kruskal–Wallis) of granulosa cells (GCs) in OGCs cultured in vitro with or without PA for 14 days. Data are presented as mean + SEM. *n* indicates the number of oocytes or OGCs examined. *n*, number of OGCs examined.

Cattle ovaries were collected from heifers (Japanese Black cows, about 24–32 months of age, 600–800 kg) at a local slaughterhouse and transported to the laboratory at approximately 25 °C in PBS containing antibiotics within 4 h. The health condition of all cows was checked by a veterinarian. Cumulus cell-oocyte complexes (COCs) and granulosa cells (GCs) were aspirated from antral follicles (AFs, 3–5 mm in diameter). Because these oocytes and GCs served as the developmental benchmark for in vitro-grown OGCs, oocytes collected from AFs were defined as in vivo-developed oocytes (Vivo oocytes), while GCs collected from AFs were used for the preparation of vivo-GCs, as described below. Oocyte–granulosa cell complexes (OGCs) were collected from EAFs on the ovary surface. As shown in Figure 1A, Thin slices of ovarian cortical tissue were obtained from the ovarian surface using a stereomicroscope (Olympus Tokyo Japan). EAFs (400–700 µm in diameter, as measured using the scale integrated into the microscope) were mechanically isolated using 21-gauge needles in IVC-medium, followed by measurement of the oocyte diameter (90–100 µm) under a digital microscope (Keyence Osaka Japan). OGCs surrounded by thick compact GCs were selected. Only OGCs derived from EAFs were subjected to in vitro growth culture, whereas OGCs collected from AFs were used as the in vivo-developed control in the experiments. For the in vitro growth culture experiments, 10 EAF-derived OGCs were cultured for 14 days in each replicate. Oocytes grown from 420 OGCs were randomly selected and used for evaluation. Oocytes grown from 300 OGCs were subjected to IVM, and randomly selected oocytes were used for evaluation. Oocytes grown from 180 OGCs were used for IVM followed by IVF. GCs of in vitro-grown OGCs were collected in 3 replicates and used for RNA-seq (60 OGCs). A total of 100 heifers (960 OGCs) were used in the present experiments.

### 2.5. Preparation of Gel Substrate Made of Polysaccharide Gel for In Vitro Oocyte Growth

The gel substrate used for OGCs incubation was prepared as described previously [9]. An equal weight ratio (0.5%) of xanthan gum (XG) and locust bean gum (LBG) (Sansho Co., Ltd., Osaka, Japan) (total 1% weight/volume) in PBS was heated in an autoclave at 120 °C for 20 min, then cooled to room temperature. Subsequently, 50 µL of the melted gel was injected into the bottom of each well of a 96-well plate (Becton, Dickinson and Company, Franklin Lakes, NJ, USA), followed by cooling, and IVG medium was placed on the gel and incubated overnight. In the experiment, the medium was removed and replaced with 200 µL of fresh IVG medium, and OGCs were cultured on the gel substrate.

### 2.6. In Vitro Growth of OGCs

The individual OGCs were transferred into wells containing 200 μL of IVG medium with either 0 or 0.01 mM PA and cultured for 14 days. Culture duration is determined by the previous report [11] and the preliminary experiment. Half of the medium was replaced with fresh medium, and antrum formation was examined after 4, 8, 10, 12, and 14 days of incubation. In this culture system, OGCs that did not form an antrum (Figure 1A) by the end of the culture period contained degenerated oocytes. Therefore, OGCs that formed an antrum were selected for the experiment. Therefore, OGCs were incubated for 14 days (under 5% CO_2_, 5% O_2_, and 90% N_2_ at 38.5 °C in the first 8 days, followed by 5% CO_2_ in air for 6 days at 38.5 °C) [12].

### 2.7. Measurement of Oocytes

Oocytes developed in vivo (vivo oocytes) and in vitro (control oocytes and 0.01 mM oocytes) were denuded of surrounding GCs. The outer diameter (including the zona pellucida) and inner diameter (excluding the zona pellucida) were measured in both the horizontal and vertical directions using a digital microscope (Keyence, Tokyo, Japan), and the average diameter was calculated [9]. The thickness of the zona pellucida was determined by subtracting the inner diameter from the outer diameter.

### 2.8. Measurement of GCs Number of OGCs

To examine the number of GCs comprising the OGCs, GCs were separated from the enclosed oocytes using a pipette and then dispersed by vigorous pipetting in Accumax Cell Dissociation Solution (Innovative Cell Technology, San Diego, CA, USA), followed by trypan blue (Nacalai, Kyoto, Japan) staining [9]. The total cell number was calculated from the concentration determined using a hemocytometer and the volume of the cell suspension. The survival rate was determined using trypan blue staining. Approximately 200 GC samples were examined in each replicate.

### 2.9. Glucose Content in Spent Culture Medium Used for IVG

On day 14, the medium was collected from individual culture wells where OGCs had formed an antrum, centrifuged (7000× *g* for 3 min) to remove cellular debris, and the concentration of glucose was measured using the LabAssay glucose kit (Fujifilm Wako Pure Chemical Corporation, Osaka, Japan). A total of 44 and 45 samples of medium were examined for the control and 0.01 mM groups, respectively. Glucose consumption was calculated by subtracting the final glucose content from the initial glucose content of the medium.

### 2.10. Preparation of GCs Derived from Large Antral Follicles

GC collected from AFs (3–5 mm in diameter) was filtered through a 70-μm cell strainer (Falcon, No 352350) and separated by gentle pipetting in Accumax (Innovative Cell Technology, San Diego, CA, USA), followed by centrifugation (300× *g* for 2 min). Survival rate of GCs derived from in vitro-developed OGCs was over 95%; however, the rate of GCs derived from AFs was approximately 70%. To select live GCs, AF-GCs were incubated in IVC-medium using a 90-mm dish for 6 h, and then live GCs were collected from the surface of the share using Accumax (Innovative Cell Technology) after centrifugation (300× *g* for 5 min). The GCs were used as in vivo GCs. Notably, although live GCs were selected, the 6 h incubation period may have affected GC gene expression profiles, and this should be considered in the interpretation of the results.

### 2.11. In Vitro Maturation and Fertilization of Oocytes

IVM and IVF were performed as previously described [10]. After 14 days of IVG, OGCs that had formed antra were selected, and oocytes surrounded by two to three GC layers were dissected from the OGCs and cultured in IVM medium for 24 h. Then, the ratio of oocytes at the metaphase II stage was examined, or the fertilization rate followed by IVF was determined. Furthermore, COCs collected from AFs were subjected to IVM and IVF, similar to in vivo oocytes. For IVF, COCs were co-incubated in IVF medium with freeze-thawed semen from a Japanese black bull for 5 h and subsequently cultured for 13 h to examine the fertilization rate. The semen was washed with a 45–60% Percoll solution (Amersham Biosciences, Uppsala, Sweden) to create a discontinuous gradient for centrifugation (800× *g* for 10 min). The final sperm concentration in the IVF medium was 1 × 10^6^ cells/mL. To evaluate the fertilization rate, oocytes were denuded of the surrounding GCs, transferred to aceto-alcohol (ethanol: acetic acid = 3:1) for 3 min, and the number of pronuclei was examined under a stereomicroscope (Olympus, Tokyo, Japan). Oocytes with two clear pronuclei at 18 h post-insemination were considered normally fertilized. IVM and IVF were conducted under 5% CO_2_ in air at 38.5 °C.

### 2.12. Measurement of Lipid Content in Oocytes and GCs

Oocytes were denuded of surrounding GCs and then fixed in 4% paraformaldehyde overnight. As reported previously [4,13], the oocytes were stained with Nile Red (10 μg/mL, Wako, Osaka, Japan) for 10 min and mounted onto glass slides with an anti-fade reagent containing DAPI (Invitrogen, Carlsbad, CA, USA). Oocytes were observed under a Leica DMI 6000B microscope using LAS AF software (DMI6000B, Leica, Wetzlar, Germany), and the fluorescence intensities of the oocytes were quantified using ImageJ software (NIH, Ver 1.53. Bethesda, MD, USA). GCs collected from OGCs or in vivo GCs were separated as described above and stained with Nile Red and Hoechst 33342. Cellular samples were mounted on slides and observed under a microscope (Keyence). The fluorescence intensity of individual cells was captured and quantified using the ImageJ software (NIH), as described above. A minimum of 120 randomly selected cells was counted in each group.

### 2.13. Mitochondrial Membrane Potential of Oocytes

As reported previously [14,15], denuded oocytes were incubated in IVC-medium containing 250 nM MitoTracker^TM^ Orange CMTMRos (for active mitochondria) and 200 nM MitoTracker^TM^ Green FM (for all mitochondria) for 15 min and observed under a fluorescence microscope (Keyence, Osaka, Japan) to capture images of equatorial region of the oocytes followed by quantification using ImageJ software (NIH, Bethesda, MD, USA).

### 2.14. Measurement of ATP in Oocytes and CGs

Individual oocytes were transported to 50 μL of distilled water, and ATP content in the oocytes was measured using the luciferase-luciferin reaction as reported previously [4]. For the measurement of ATP in GCs, GCs were transferred into 100 μL of distilled water. After repeated freeze-thaw cycles, 50 μL of the suspension was subjected to ATP measurement, and the remaining 50 μL was mixed with 2× DNA extraction buffer, as described below for PCR targeting a single-copy gene. The ATP content was normalized to the copy number of the corresponding GC samples.

### 2.15. Measurement of Mitochondrial DNA Copy Number (Mt-Cn) and Number of One Copy Genes

Measurements were performed as previously described [10]. In brief, DNA was extracted in extraction buffer (Tris-HCl containing 20 mM Nonidet P-40, 0.9% Tween 20, and 0.4 mg/mL proteinase K) by heating at 55 °C for 30 min and at 98 °C for 10 min. PCR program for both mt-cn and single-copy gene is 95°C for 3 min, followed by 39 cycles of 97 °C for 6 s and 60 °C for 10 s. Primers used for mt-cn measurement were Forward, 5′-ACCCCTTGTACCTTTTGCAT-3′ and Reverse, 5′-TCTGGTTTCGGGCTGCTTAG-3′ (81 bp, NC_037343.1, mitochondrial genome), and those used for single-copy gene were Forward, 5′-TTCCACTCTGCACAGTAGCG-3′ and Reverse,5′-CCCTTACTGGTTGTGGCACT-3′ (83 bp, NC_037334.1, 1-copy gene). Serial dilutions were prepared using plasmids cloned from the corresponding PCR products and were sequenced. The copy numbers in the standards were calculated using the concentration and Avogadro’s number.

### 2.16. RNA-Sequence (RNA-Seq) of GCs

GCs collected from at least three antrum-forming OGCs after 14 days of IVG and in vivo GCs derived from at least 10 cows were used. Three independent samples were prepared from different ovarian series.

Total RNA was extracted using the RNAqueous TM-Micro Kit (Thermo Fisher Scientific, Waltham, MA, USA), and the RNA quality and concentration were assessed using a 2100 Bioanalyzer (Agilent Technologies, Santa Clara, CA, USA). The average RIN value was 9.2 ± 0.2. An RNA-seq library was prepared using the NEBNext UltraExpress RNA Library Prep Kit (#E3330L; New England Biolabs, Ipswich, MA, USA). Library concentrations were determined using the Agilent TapeStation D1000 kit and TapeStation 4200 (Agilent Technologies). Library concentrations were reassessed using a Kapa Library Quantification Kit (Kapa Biosystems, Wilmington, MA, USA). The multiplexed samples were sequenced in single-read 100-bp reads using the NextSeq1000 system (Illumina, San Diego, CA, USA). Raw data were generated using the bcl2fastq2 software (Illumina) according to the manufacturer’s instructions. Adapter sequences, ambiguous nucleotides, and low-quality reads were removed before downstream analysis. The filtered reads were aligned to the Bos taurus reference genome (ARS-UCD1.2.109), and read counts were obtained. Gene expression levels were calculated as transcripts per kilobase million (TPM).

#### Differentially Expressed Genes (DEGs)

Differentially expressed genes (DEGs) were identified using transcriptomic analysis tools in the CLC Genomics Workbench with a threshold of *p* < 0.05. For pathway analysis, DEGs were further filtered using fold-change thresholds of ≥1.5 for the PA vs control comparison and ≥5 for the PA vs in vivo comparison. These different thresholds were applied because the total number of DEGs identified at *p* < 0.05 differed substantially between the comparisons (1542 genes for PA vs control and 10,256 genes for PA vs in vivo). Applying the same threshold to both comparisons resulted in either excessive filtering or inclusion of an excessively large number of genes, which reduced the specificity of pathway enrichment analysis. Functional enrichment analysis of the DEGs was performed based on Kyoto Encyclopedia of Genes and Genomes (KEGG) pathways using the DAVID functional annotation tool (https://davidbioinformatics.nih.gov/, accessed on 29 May 2026), with Bos taurus as the background species. *p*-values were calculated using Fisher’s exact test. RNA-seq data were deposited in the DNA Data Bank of Japan (DDBJ) BioProject database under the accession number PRJDB40521.

K-medoids clustering was performed using DEGs (*p* < 0.05) between the PA and control groups, and the gene expression profiles of AF-derived GCs (vivo GCs) were analyzed using the CLC Genomics Workbench. The DEGs were clustered into eight groups. Based on these clusters, three gene categories were defined: Group A, in which PA shifted gene expression toward the in vivo GC profile; Group C, in which PA shifted gene expression away from the in vivo GC profile; and Group B, with intermediate changes (Figure 1B). All DEGs, gene groups, pathways, and lists of genes associated with the pathways of interest are presented in Tables S1–S14.

### 2.17. Statistical Analysis

All data were analyzed using the Shapiro–Wilk test. Three parametric datasets were compared using one-way ANOVA, followed by Fisher’s exact test. Two parametric data sets were analyzed using the Student’s *t*-test. Three non-parametric data sets were analyzed using the Kruskal–Wallis test, followed by the Steel–Dwass test. The rates of nuclear maturation and fertilization were analyzed using a χ^2^ test. Statistical significance was set at *p* < 0.05, were considered significant.

## 3. Results

### 3.1. PA Improves Quality of Oocytes Grown In Vitro

**Figure 2:**
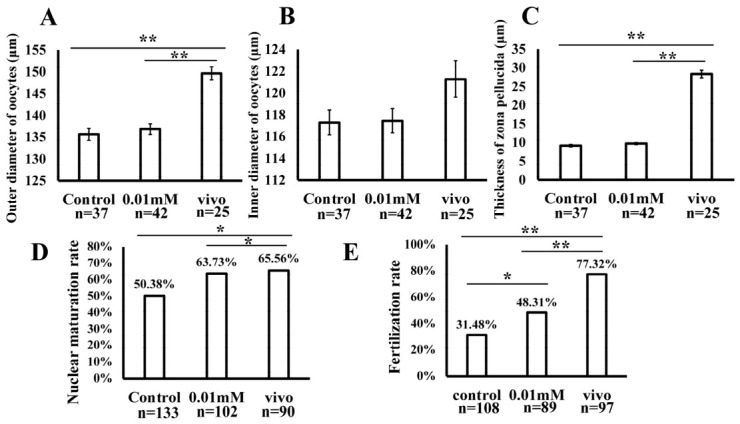
Characteristics of oocyte–granulosa cell complexes (OGCs) cultured with or without PA and those derived from antral follicles (Vivo group). Outer diameter of oocyte (one-way ANOVA) and inner diameter of ooplasm (Kruskal–Wallis) and thickness of zona pellucida (one-way ANOVA) (**A**–**C**). Nuclear maturation rate (metaphase 2) of oocytes following in vitro maturation (χ^2^ test) (**D**) and fertilization rate after in vitro fertilization treatment (χ^2^ test) (**E**). Data are presented as the mean ± standard error of the mean. *n* indicates the number of oocytes or OGCs. *, *p* < 0.05, **, *p* < 0.01. *n*, number of oocytes or GCs examined.

PA did not affect the rate of antrum formation, the total number of GCs constituting the OGCs, their survival rate, oocyte diameter, or ZP thickness (Figure 1C–E and Figure 2A,B). However, the rates of nuclear maturation following IVM and normal fertilization after IVF were improved by PA supplementation. (Figure 2C,D). Compared to the parameters of in vivo oocytes, oocyte size, zona pellucida thickness, and fertilization rate were lower for oocytes grown in vitro, regardless of the culture conditions. (Figure 2A,B,D).

### 3.2. Characteristics of Oocytes and GCs After In Vitro Development

#### 3.2.1. Lipid Content

**Figure 3:**
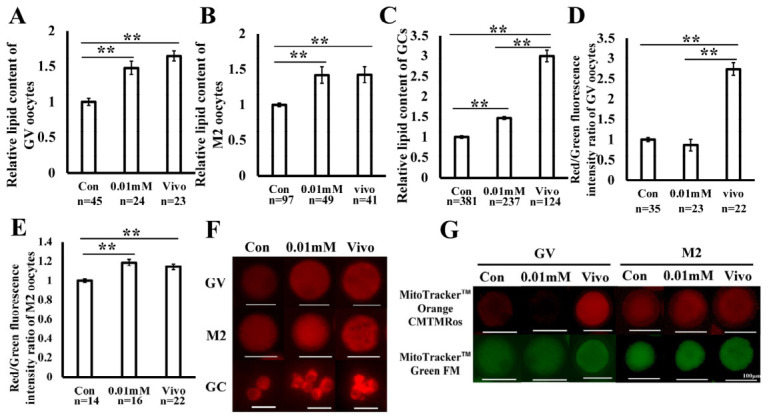
Lipid content and mitochondrial membrane potential in oocytes grown in vitro with (0.01 mM) or without (Con) propionic acid and those derived from antral follicles (vivo group) and corresponding granulosa cells. Lipid content in oocytes before (one-way ANOVA) and after in vitro maturation (one-way ANOVA) (**A**,**B**), respectively, and representative pictures (**F**) Lipid content in the granulosa cells surrounding oocytes grown in vitro and in vivo (one-way ANOVA) (**C**,**F**) Ratio of active (Red) per whole mitochondria (Green) in oocytes before (Shapiro–Wilk test) and after in vitro maturation (one-way ANOVA) (**D**,**E**) and representative pictures (**G**). The data are presented as the mean ± standard error of the mean. The value of Control is defined as 1.0 in (**A**–**D**). *n*, number of oocytes or GCs examined. ** *p* < 0.01. The scale bar represents 100 (for oocytes) and 20 μm (for granulosa cells). *n*, number of oocytes examined.

PA supplementation increased the lipid content in oocytes grown in vitro, and the levels reached those observed in vivo oocytes (Figure 3A,F), and this difference persisted after IVM (Figure 3B,F). Supplementation of the culture medium with PA also increased the lipid content of GCs, although the levels remained lower than those in GCs derived from AFs (vivo GCs; Figure 3C,F).

#### 3.2.2. Mitochondrial Membrane Potential

PA supplementation did not affect the mitochondrial membrane potential of oocytes before IVM (Figure 3D,G). However, after IVM, the mitochondrial membrane potential in the PA group increased to levels comparable to those observed in vivo (Figure 3E,G).

#### 3.2.3. Adenosine Triphosphate (ATP) Content

**Figure 4:**
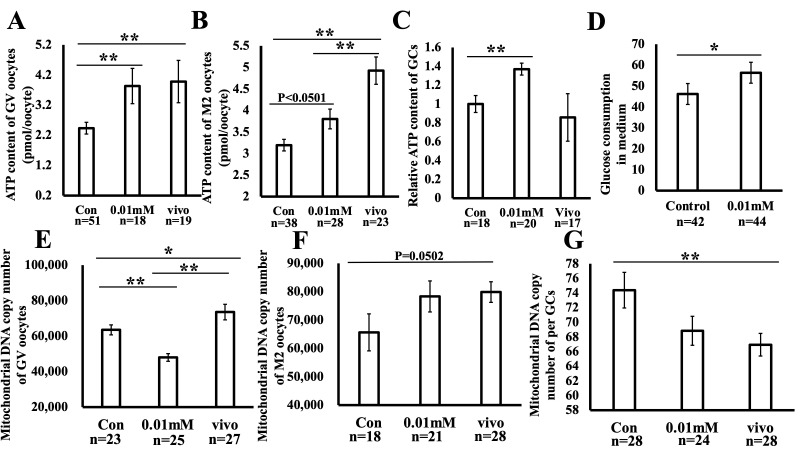
ATP, glucose consumption, and mitochondrial DNA copy number (mt-cn) in oocytes and granulosa cells (GCs). Oocyte–granulosa cell complexes (OGCs) incubated with (0.01 mM) or without (Con) PA for 14 days and OGCs collected from antral follicles (Vivo group) were examined. ATP content in oocytes before (one-way ANOVA) (**A**) and after maturation (one-way ANOVA) (**B**) and granulosa cells before maturation (Shapiro–Wilk test) (**C**). Glucose consumption of the OGCs (Student’s *t*-test) (**D**). Mt-cn in oocytes before (Student’s *t*-test) (**E**) and after maturation (Student’s *t*-test) (**F**) and granulosa cells of oocytes before maturation (Student’s *t*-test) (**G**). Data are presented as the mean ± standard error of the mean. Value of Control is defined as 1.0 (**A**,**C**). *n*, number of oocytes or GCs examined. * *p* < 0.05, ** *p* < 0.01. *n*, number of oocytes examined.

PA supplementation elevated the ATP content in oocytes, similar to that of vivo oocytes prior to IVM (Figure 4A). However, after IVM, ATP levels were significantly higher only for in vivo oocytes compared with those in in vitro-developed counterparts (Figure 4B). The ATP content in the GC was higher in the propionic group than in the control group (Figure 4C). PA supplementation was also upregulated by consumption (Figure 4D).

#### 3.2.4. Mitochondrial Copy Number (Mt-Cn)

Mt-cn levels were significantly lower in in vitro-grown oocytes, with the lowest levels observed in the PA group (Figure 4E). However, no significant differences were detected among the three groups after IVM. Among the GCs derived from antral follicles (vivo GCs), Mt-cn expression was the lowest among the three groups (Figure 4G).

### 3.3. RNA-Seq

RNA-seq revealed 1636 DEGs between the PA group and control (*p* < 0.05) and 8909 (*p* < 0.01) between the PA group vs In vivo group. From the DEGs, 740 DEGs between the PA group vs control: 740 DEGs (*p* < 0.05, fold change ±1.5; Table S1) and 2042 DEGs between the PA group vs In vivo group (*p* < 0.01, fold change ±5; Table S2) were subjected to functional annotation. PA—control DEGs were associated with the cytoskeleton in muscle cells, cell adhesion, and the PI3K-Akt signaling pathway (Table 1 and Table S3). PA group vs In vivo DEGs were associated with the cytoskeleton in muscle cells, focal adhesion, and PI3K-Akt signaling (Table 1 and Table S4). Several pathways were shared by the two DEGs groups; 23 of 28 DEGs associated with the cytoskeleton in muscle cells were downregulated in PA groups compared with the control, and 58 of 69 genes were expressed more in vivo GCs than in propionic groups. The same was observed for the PI3K-Akt signaling pathway, where 23 of 30 DEGs associated with the pathway were downregulated in PA groups compared with the control, and 66 of 80 genes were expressed at higher levels in vivo GCs compared with propionic groups (Tables S5–S8).

To gain more detailed insight into metabolism, genes related to lipid, glucose, and hypoxic responses were compared between the control and propionic groups. Regarding lipid metabolism, nine of the 87 genes were affected (Table S9), including downregulation of CD36 (lipid uptake). Among the 26 glycolysis-related genes, nine (*SLC2A3*, *PFKL*, *ALDOA*, *GAPDH*, *ENO1*, *PDK1*, *EGLN3*, and *VEGFA*) were upregulated, including two hypoxia-related genes (*EGLN3* and *VEGFA*) (Table 2 and Table S10).

Contrary to the comparison between PA groups and control, many genes related to lipid metabolism and glycolysis were differentially expressed between the PA group and in vivo GCs, especially lipid intake and transport-related genes (*CD36*, *SLC27A2/4*, *FABP4/5*) were upregulated in the in vivo group, whereas lipid synthesis-related genes (*ACACB*, *FASN*, *ME1*, *FADS1/2*, and *SCD*) were downregulated, and the DEGs contain other lipid metabolism-related genes (*PNPLA2*, *LIPE*, *PLIN1/2/3*, and *CIDEA/B/C*) (Table 2 and Tables S9 and S10). Furthermore, of the 26 selected glycolysis genes, 19 genes were expressed at low levels in in vivo GCs (Table S10). In addition, hypoxia-related genes, such as *ARNT*, *EGLN3*, *VHL*, *VEGFA*, *LDHA*, *BNIP3*, *PDK1*, and *CA9*, were expressed at lower levels in vivo in GCs. Genes belonging to Group A (Figure 1B and Table S11), as determined by K-medoid clustering and gene expression profiles of the in vivo group, were associated with Alzheimer’s disease and oxidative phosphorylation (Table 1 and Table S13). In contrast, genes belonging to Group C (Table S12) were associated with the MAPK signaling pathway, thyroid hormone synthesis, and the PI3K-Akt signaling pathway (Table 1 and Table S14).

## 4. Discussion

The present study demonstrates that PA improves the quality of oocytes grown in vitro by enhancing glucose utilization and the energy status of enclosed oocytes. Even though the improvement, the quality of the in vitro-grown oocytes with PA was still lower than that of in vivo-grown oocytes. RNA-seq analysis of GCs revealed that some gene groups were distinctly affected by PA compared with those in control and in vivo GCs.

In ruminants, the blood contains high concentrations of acetic acid and PA, and these short-chain fatty acids may influence the metabolism of GCs and oocytes. In the present study, only PA was added to the culture medium; therefore, the interpretation of our findings should be limited to its specific effects. Although a few studies have investigated the effects of low concentrations of fatty acids on oocyte maturation [4,16,17], this is the first study to investigate the effect of PA on oocyte growth.

PA affects oocyte characteristics, maturation, and fertilization abilities.

The diameter of oocytes is a common marker of oocyte growth, but the present study did not alter oocyte size; instead, it improved oocyte maturation and fertilization ability, indicating enhanced cytoplasmic maturation. Bovine oocytes contain a large amount of lipid in the cytoplasm [18,19,20], and the lipids in oocytes serve as energy substrates for oocyte maturation [21,22,23,24,25]. In addition, the number of GCs and glucose consumption are correlated with lipid and ATP content in oocytes [21,26]. The present study showed that supplementation with PA increased the lipid and ATP contents in both oocytes and GCs. Considering that granulosa cells support oocyte metabolism through gap junctions [27,28,29,30], it has been suggested that a part of the effect of PA is derived from increasing energy levels in GCs. GCs support oocyte energy status via glycolysis and transport of bioactive molecules into oocytes [31,32]. Consistently with previous reports that PA increased glucose utilization in C2C12 myotubes and muscle [33,34], the present study showed that glucose uptake by OGCs increased by PA supplementation, and RNA-seq revealed that PA increased the expression of glycolytic genes. Glycolytic activity is associated with hypoxic conditions [35,36], and the upregulation of Hypoxia-inducible factor 1 (HIF1) target genes *EGLN3*, *PDK1*, *BNIP3*, *CA9*, *ENO1*, *ALDOA*, and *VEGFA* in the PA-treated GCs suggests PA may induce pseudo-hypoxic conditions in GCs and increase glucose utilization in GCs, thereby improving the energy status of enclosed oocytes. This notion should be examined by further studies. Notably, the mitochondrial number was lower, and the lipid content was higher in the in vivo group than in the in vitro-cultured GCs. It has been reported that either FF or fatty acid mix increases lipid content in oocytes and cumulus cells [37,38]. In addition, low O_2_ conditions activate glycolysis and reduce mitochondria in bovine GCs [35]. Furthermore, RNA-seq analysis revealed that genes associated with lipid uptake and accumulation were highly expressed in in vivo GCs. Collectively, the RNA-seq analysis revealed distinct lipid metabolism-related pathways in in vivo GCs, suggesting that the in vivo follicular environment may influence the metabolic characteristics of GCs. However, because the present study did not directly assess follicular fluid composition or other metabolic parameters, further studies are required to clarify the underlying mechanisms.

Low mitochondrial membrane potential and mitochondrial number in in vitro-cultured oocytes compared with in vivo oocytes have previously been reported in mice [39]. Consistently, we also observed lower mitochondrial membrane potential and mitochondrial number in in vitro-grown oocytes in the present study. However, the difference in mitochondrial number between in vitro- and in vivo-grown oocytes was reduced after maturation. Since both mitochondrial membrane potential and mitochondrial number have been reported to affect oocyte maturation and developmental competence [40,41,42], these findings suggest that mitochondrial function in in vitro-grown oocytes remains immature and that the extent of this immaturity depends on in vitro culture conditions. Functional annotation analysis of DEGs revealed that similar functional categories were enriched among DEGs identified between the control and PA groups and those identified between PA-treated and in vivo GCs. These findings indicate that, although PA improved oocyte quality, it also exerted distinct effects on GCs. Based on K-medoids clustering analysis and the gene expression profiles of in vivo GCs, two gene clusters (Groups A and B) were selected. Genes in Group A were associated with oxidative phosphorylation (OXPHOS), suggesting that PA influences mitochondrial function in a manner similar to that observed in vivo. In contrast, genes in Group B were associated with PI3K–Akt signaling. This finding was consistent with the functional annotation results of DEGs between the PA and in vivo groups, which also showed enrichment of the PI3K–Akt signaling pathway. In addition, genes related to cell adhesion, cytoskeletal organization, and PI3K–Akt signaling appeared to be specifically affected by PA. However, these effects may be moderated under in vivo conditions, where follicular fluid contains various types of fatty acids. Further studies investigating the effects of other short-chain fatty acids on oocyte development are required. It is noteworthy that the present vivo GC group potentially included GCs derived from AFs undergoing atresia, because follicular status at the 3–5 mm stage is difficult to determine morphologically. Nevertheless, AF-derived oocytes are widely used as the physiological reference for bovine embryo production and therefore serve as the practical in vivo benchmark in the present study. More precisely selected healthy AFs would provide a more ideal reference for evaluating the quality of in vitro-grown oocytes.

In conclusion, PA supplementation improved the in vitro growth of bovine oocytes, likely through enhancement of cellular energy metabolism. However, PA supplementation alone was insufficient to fully reproduce the characteristics of in vivo-grown oocytes.

## Figures and Tables

**Table 1:** KEGG pathways associated with differentially expressed genes.

Pathway Name	Count	*p*-Value
Control and propionic acid group		
Cytoskeleton in muscle cells	28	0.000
PI3K-Akt signaling pathway	30	0.000
MAPK signaling pathway	24	0.000
Focal adhesion	18	0.000
Cell adhesion molecules	16	0.001
Pathways in cancer	34	0.001
TGF-beta signaling pathway	12	0.001
Complement and coagulation cascades	11	0.001
ECM-receptor interaction	10	0.003
Propionic acid and the in vivo group		
Cytoskeleton in muscle cells	69	0.000
Focal adhesion	55	0.000
PI3K-Akt signaling pathway	80	0.000
Complement and coagulation cascades	33	0.000
Pathways in cancer	100	0.000
Proteoglycans in cancer	50	0.000
Staphylococcus aureus infection	33	0.000
ECM-receptor interaction	30	0.000
Cell adhesion molecules	41	0.000
K-medoid Group A		
Alzheimer disease	18	0.000
Chemical carcinogenesis—reactive oxygen species	13	0.000
Oxidative phosphorylation	10	0.000
Huntington disease	14	0.000
Pathways of neurodegeneration—multiple diseases	17	0.000
K-medoid Group C		
Metabolic pathways	49	0.007
MAPK signaling pathway	14	0.009
PI3K-Akt signaling pathway	16	0.015
Thyroid hormone synthesis	6	0.020
Calcium signaling pathway	12	0.020

**Table 2:** Expression value of genes of interest.

Name	Gene Name	Propionic/Control		Propionic/Vivo	
		Fold Change	*p*-Value	Fold Change	*p*-Value
Lipid metabolism	*CD36*	−1.787	0.000	−1.698	0.000
	*SLC27A2*	2.207	0.019	−1.750	0.024
	*SLC27A4*	−1.368	0.048	−1.702	0.000
	*FABP4*	#N/A	#N/A	−1016.740	0.000
	*FABP5*	−1.453	0.076	−7.219	0.000
	*ACACB*	−1.025	0.721	1.885	0.000
	*FASN*	−1.144	0.217	1.505	0.000
	*ME1*	−1.016	0.877	1.431	0.000
	*FADS1*	1.124	0.296	4.222	0.000
	*FADS2*	−1.075	0.346	3.873	0.000
	*SCD*	1.234	0.025	5.900	0.000
	*PNPLA2*	−1.323	0.049	−1.598	0.000
	*MGLL*	−2.770	0.035	−7.014	0.000
	*LIPE*	−1.108	0.620	2.759	0.000
	*PLIN1*	1.063	0.831	2.200	0.003
	*PLIN2*	−1.078	0.425	−28.192	0.000
	*PLIN3*	1.118	0.277	−1.738	0.000
	*CIDEA*	1.113	0.835	−2.651	0.007
	*CIDEB*	1.458	0.365	2.371	0.018
	*CIDEC*	−6.332	0.296	−54.295	0.018
Glycolysis	*SLC2A3* *	1.347	0.018	5.386	0.000
	*PFKL*	1.206	0.041	4.638	0.000
	*ALDOA* *	1.203	0.048	2.987	0.000
	*GAPDH*	1.310	0.005	3.537	0.000
	*ENO1* *	1.313	0.011	6.328	0.000
	*PDK1* *	1.285	0.022	7.501	0.000
	*SLC19A3*	1.301	0.036	13.117	0.000
	*EGLN3* *	1.515	0.000	22.277	0.000
	*VEGFA* *	1.846	0.000	32.049	0.000
hypoxia	*ARNT*	1.121	0.152	1.222	0.006
	*VHL*	1.220	0.059	1.982	0.000
	*LDHA*	1.193	0.091	4.724	0.000
	*BNIP3*	1.384	0.004	9.497	0.000
	*PGK1*	1.195	0.077	2.218	0.000
	*CA9*	2.460	0.000	−7.118	0.000
	*SLC16A3*	1.301	0.036	19.233	0.000

* hypoxia response genes. #N/A Not Applicable.

## Data Availability

The data (raw FASTQ file format RNA-seq data) presented in this study are openly available from the DNA Data Bank of Japan with links to the PRJDB40521 in the DNA Data Bank of Japan (DDBJ) BioProject database. All data used in this study are submitted as supplementary tables, and additional data underlying this study will be shared using the URL: https://x.gd/9KdF7 (access date: 29 May 2026).

## Supplementary Materials

The following supporting information can be downloaded at: https://www.mdpi.com/article/10.3390/vetsci13060533/s1, Table S1: The DEGs of Control vs PA group (*p* < 0.05, fold change ±1.5); Table S2: The DEGs of PA group vs In vivo group (*p* < 0.01, fold change ±5); Table S3: The KEGG pathway analyses of Control vs PA group; Table S4: The KEGG pathway analyses of PA group vs In vivo group; Table S5: List of several pathways shared from the two DEG groups; Table S6: The DEGs of Cytoskeleton in muscle cells; Table S7: The DEGs of PI3K-Akt signaling pathway; Table S8: The DEGs of Focal adhesion; Table S9: The DEGs of Lipid metabolism; Table S10: The DEGs of Glycolysis; Table S11: The DEGs of Genes belonging to group A by K-medoid clustering; Table S12: The DEGs of Genes belonging to Group B by K-medoid clustering. Table S13: The DEGs of Genes belonging to Group C by K-medoid clustering; Table S14: The KEGG pathway analyses of Genes belonging to group A by K-medoid clustering; Table S15: The KEGG pathway analysis of Genes belonging to Group B by K-medoid clustering. Table S16: The KEGG pathway analyses of Genes belonging to Group C by K-medoid clustering.

## Abbreviations

AF/AFs	Antral follicle(s)
ATP	Adenosine triphosphate
CCs	Three-letter acronym
COCs	Linear dichroism
DAPI	4′,6 diamidino 2 phenylindole
DAVID	Database for Annotation, Visualization, and Integrated Discovery
DDBJ	DNA Data Bank of Japan
DEGs	Differentially expressed genes
EAFs	Early antral follicles
EGF	Epidermal growth factor
FF	Follicular fluid
FCS	Fetal calf serum
GCs	Granulosa cells
HIF1	Hypoxia-inducible factor 1
IVC	In vitro culture
IVF	In vitro fertilization
IVG	In vitro growth
IVM	In vitro maturation
KEGG	Kyoto Encyclopedia of Genes and Genomes
LBG	Locust bean gum
MAPK	Mitogen-activated protein kinase
MMP	Mitochondrial membrane potential
mt-cn	Mitochondrial DNA copy number
OGCs	Oocyte–granulosa cell complexes
OXPHOS	Oxidative phosphorylation
PA	Propionic acid
PBS	Phosphate-buffered saline
PCR	Polymerase chain reaction
PI3K-Akt	Phosphoinositide 3-kinase-Akt signaling pathway
RNA-seq	RNA sequencing
SOF	Synthetic oviduct fluid
TCM-199	Tissue culture medium 199
TPM	Transcripts per kilobase million
VFAs	Volatile fatty acids
XG	Xanthan gum
